# A Global Public Goods Approach to the Health of Migrants

**DOI:** 10.1093/phe/phv013

**Published:** 2015-06-01

**Authors:** Heather Widdows, Herjeet Marway

**Affiliations:** University of Birmingham

## Abstract

This paper explores a global public goods approach to the health of migrants. It suggests that this approach establishes that there are a number of health goods which must be provided to migrants not because these are theirs by right (although this may independently be the case), but because these goods are primary goods which fit the threefold criteria of global public goods. There are two key advantages to this approach: first, it is non-confrontational and non-oppositional, and second, it provides self-interested arguments to provide at least some health goods to migrants and thus appeals to those little moved by rights-based arguments.

This paper adopts a global public goods approach tothe health of migrants. This approach is unusual, as debates about migrants and what is owed to them, in general, are largely rights-based. This paper will briefly outline the current rights-based nature of such debates and suggests that alternative approaches might be useful. We begin by noting the dominance of rights language in the current debate and suggest that this is confrontational and oppositional, so motivating the seeking of alternative approaches. We present a global public goods approach building on previous work and consider what, if anything, such an approach would deliver in terms of migrant health (Widdows and Cordell, 2011; Widdows, 2013; Widdows and West-Oram, 2013).^1^ To this end, we define public goods using three key criteria, show how these apply using the examples of the environment and antibiotic efficacy and then apply this model to the health of migrants. This approach might, at first glance, seem unlikely to deliver, as it is not obvious why one needs to protect migrant health to protect the health of all. But, while not delivering all the goods of health and healthcare, one might wish it will deliver some, and some significant health goods. We argue that there are two key advantages to our approach: first, it is non-confrontational and non-oppositional, so may be useful in surmounting the current impasse which assumes that one group can only benefit at the expense of another, and second, as a result, it may convince those who have little interest in the rights of migrants to support the provision of health goods to them. Admittedly, this is a tentative paper which merely begins to explore a different conceptual approach.

## Seeking New Frameworks

Much of the work on the health of migrants, and on the rights of and the duties to migrants, uses the human rights framework to make justice claims, to delineate the rights of migrants and the duties owed to all individuals. Too often, and to caricature, this debate collapses into a conflict between the rights of some individuals and the rights of others. This is true of many of the discourses which surround migration, and which the debate about the health of migrants draws upon. Rights language is dominant in discussions around defending the rights of immigrants and immigration policy. For instance, the International Convention on the Protection of the Rights of All Migrant Workers and Members of Their Families (2003) seeks to protect the basic freedoms of all (documented and undocumented) migrants, a proposal which is based on realizing the individual rights that all persons hold under the Universal Declaration of Human Rights (1948) (UN, 1990).^2^ Similarly, there is discussion on the links and tensions between fundamental, natural and human rights—all of which focus on individual rights—and on the extent to which immigration policies might be liberalized (Ghoshray, 2006–2007). Theorists have tended to compare the interests of one group of people (migrants) against those of another group (low-skilled, low-paid citizens) and have suggested that more open migration policies will exacerbate inequalities for the poorest nationals (Borjas, 2001; Cafaro, 2008). In this regard, and in general, the immigration debate is often couched in the terms of right versus right (Teitelbaum, 1980). In these complex and competing narratives, the rights of some individuals are presented as trumping the rights of others, and it is assumed that granting rights to one group of individuals will be at the expense of the rights of another group.

Furthermore, such discourses about migrants are often highly rhetorical and emotional. For instance, poor migrants who are forced to become such, either as refugees fleeing from conflict zones or economic migrants seeking to escape grinding poverty, are caricatured (especially by those who are anti-migration) as ‘flooding countries’ and taking jobs.^3^ Other migrants, particularly highly qualified migrants—colloquially called the brain drain—are criticized for leaving their countries of origin. Certainly such movements cause difficulties in developing countries, evidenced clearly in the low numbers of health professionals who remain in the developing world. But, conversely, remittances are an important source of income for such countries.^4^ Such emotional language makes claims for the rights of migrants controversial, especially if rights language is used, as this language tends to imply both confrontation and opposition.

It is the individual and confrontational nature of rights language which leads us, somewhat tentatively, to approach the issue of the health of migrants from a different perspective, one which is not rights-based, and which focuses on communal goods rather than individual goods.^5^ This is not to suggest that individual approaches should be abandoned; on the contrary, we consider many of these to be strong and useful, and as global ethicists, we endorse rights and duties for and to all individuals globally. However, while individually focused theories are crucial to global justice theorizing and individuals must be regarded as the primary locus of moral concern, overly individualist theories fail to recognize key goods and harms, because theories determine a priori which goods and harms can be recognized and which cannot (Widdows and West-Oram, 2013). Our alternative approach is not intended to replace rights-based approaches, but to complement and to be used alongside other approaches.

## Defining Global Public Goods

In this paper, we focus on global public goods, rather than public goods in general. Definitions of (global) public goods are contentious; some are descriptive and some are normative. Adopting a descriptive definition supposedly avoids value-laden claims and merely points to goods which cannot be other than public, while normative descriptions make claims that such goods have a status which merits protection. Our contention is that descriptive definitions imply a normative definition in the case of *global* public goods; why this is so will become clear as we discuss the nature of these goods.

Let us begin by describing public goods in general, as opposed to global public goods. Public goods are enjoyed collectively and, as such, are non-rivalrous (in that their use by one does not prevent their use by another) (Kaul *et al.*, 1999a), lack excludability (they are inclusive and available to all) and require collective management and maintenance. Examples of public goods include traffic lights (Kaul *et al.*, 1999a), laws (Widdows and Cordell, 2011) and education (Kaul *et al.*, 1999b; Sen, 1999). Domestic public goods are enjoyed collectively within a geographical location or as part of a community and are characterized by being beneficial to those who have access to them, as well as being collectively protected and sustained. This description—especially at the non-global level—is purely descriptive. For instance, to say that to obey laws or contribute to street lighting is a public good, which can only be communally and publically maintained, is to describe the good. This does not necessarily imply a normative claim that such goods *should* be protected in all circumstances and beyond other goods. Indeed, it is not hard to imagine instances where these goods should not be maintained: there are instances where laws can justifiably be broken and street-lighting dimmed (for instance in blackouts or for celebrations). Such local goods might contribute to well-being, but they are open to change and can be less important than other goods.

When it comes to global public goods in addition to the descriptive claims—of collective sustainability, non-excludability and so on—we add further descriptive claims upon which we invoke a normative claim. Global public goods, in contrast to other public goods, are goods which require *all* individuals to behave in certain ways if they are to be sustained (descriptive claim). More importantly, in this category are *only* those public goods which if not sustained would dramatically harm the well-being of *all* individuals (another descriptive claim). These descriptive claims define goods which are crucial to protect (because the harms which follow if they are not are so severe) and which require action by all, and so result in a normative assertion that they *should* be protected. Accordingly, such global public goods should be treated as ‘primary goods’ and should be protected legally and in policy and at all levels regardless of the wishes of individuals or states. To break this down, according to this definition of global public goods, three criteria must be met:

- First, if the global public good is not protected then *all* individuals (current and future) will be exposed to significant harm (and often will actually suffer harm, harms preventable by the protection of the good),
- Second, the global public good cannot be protected without collective action (nor can the resulting harms be prevented without collective action),

If these two descriptive criteria are met then we argue that a—normative—claim is implied, that:

- Third, a global public good which meets the descriptive criteria is a primary good which *should* be protected to prevent significant harms to all individuals and accordingly states and/or individuals cannot be allowed to choose to neglect this good.^6^

If this reasoning holds, the normative claim follows upon the descriptive claims, in that if the first two criteria are correct, then one has strong reasons for accepting the third, as only if one accepts the third can the good (established as primary by criteria one and two) be systematically protected. If the good really is a primary good—failure to protect it results in exposure of all individuals to significant harm and it can only be protected by collective action—then the third criteria *should* apply. In practice, the normative claim may not be recognized or respected—and we will explore this—even though it reasonably follows from the first two criteria. Of course if any of the criteria can be shown not to apply—for instance, that the harm is not significant or that collective action is not required to protect the good—then the claim will, of course, be undermined. But, this would not be to deny the normative claim, but rather to deny that the good in question really is a primary good of the type under discussion. To illustrate, let us consider the environment, the archetypal global public good and antibiotic efficacy, which we have previously argued should also be considered in this category.

## The Environment

The environment is collectively enjoyed by all, it is non-rivalrous and non-excludable and requires collective maintenance. In terms of the first criterion, if the environment is not protected, then *all* individuals (current and future) will be exposed to significant harm. Likely harms include those which follow from increases in sea level (Barnett and Adger, 2003), coastal and habitat erosion (Feagin *et al.*, 2005), species extinction (Thomas *et al.*, 2004), extreme weather events (McMichael *et al.*, 1996), exacerbated health risks (McMichael and Haines, 1997; Haines *et al.*, 2006), greater movement of people (Reuveny, 2007) and increased risks of conflict (Barnett and Adger, 2007). Already people are suffering as a result of climate change; for instance, increased flooding is documented in a number of African cities (Douglas *et al.*, 2008) and extreme weather events have already been experienced (for instance, the 2003 European heat waves and the 2004 and 2005 Atlantic hurricane seasons; Van Aalst 2006)^7^ Accordingly, current and future individuals are likely to actually suffer harm, harms which would have been prevented had the environment been adequately protected. Likewise, the second criterion is met, as the environment cannot be protected without collective action (nor can the resulting harms be prevented without collective action). Some individuals cannot continue to engage in environment-harming actions (from air travel to the burning of fossil fuels) if any are to avoid harm. So the first and second descriptive criteria are met.

### Efficacy of Antibiotics^8^

A parallel argument can be made for the efficacy of antibiotics. First, if antibiotic efficacy is not protected, then *all* individuals (current and future) will be exposed to significant harm (and often will actually suffer harm, harms preventable by the protection of the good). The harms of antibiotic resistance (the failure to protect antibiotic efficacy) are significant. It is possible we will return to a pre-antibiotic era where common infectious diseases again become lethal.^9^ Pathogens which are resistant to antibiotics include multi- or extremely drug-resistant tuberculosis (Ormerod, 2005), methicillin-resistant *Staphylococcus aureus* (Cosgrove, 2006) and multidrug-resistant plague (Welch *et al.*, 2007). The extent of these threats is such that on World Health Day 2011, the World Health Organization released a set of policy proposals to address antibiotic resistance (World Health Organization, 2012) and stated that ‘[t]he world is on the brink of losing these miracle cures’ (Chan, 2011). More recently, the Government of the United Kingdom hosted an international event to discuss the problem of drug resistance (UK Department of Health, 2013). Accordingly, the harms which flow from the failure to protect antibiotic efficacy are extreme and immanent.

Second, the good of antibiotic efficacy cannot be protected without collective action (nor can the resulting harms be prevented without collective action). In the current model, the use of antibiotics is largely regarded as a private issue, or one which is a matter for market forces. Accordingly, the wider consequences of inappropriate, inefficient and over- or underuse have been largely ignored (Cars *et al.*, 2008; Olivier *et al.*, 2010).^10^ In the developed world, antibiotics are essentially commodities. Antibiotics are used by those who can afford them, by consumers who are either using them as patients or for food production or agri-businesses. Patterns of use in the developing world also contribute to the erosion of the good, for complex and understandable reasons. The poor, typically in developing countries, are often unable to afford full courses of drugs. This leads to the sharing of medicines and stockpiling ‘excess doses’. As a result, efficacy decreases and resistant diseases increase. Collective action is required, for while antibiotic resistance is an inevitable consequence of any use of antibiotics, the harms could be reduced and the rapid rise of antibiotic resistance slowed significantly with collective action, so meeting the second criterion.

In both cases, of the environment and antibiotic resistance, then the first and second criteria are met and the third, normative criteria, follows from these, in the sense that if the first two are met, it would be unreasonable not to introduce policy which requires global public goods to be systematically protected. Given this then, the identification of a global public good will generate obligations on individuals, states and globally and corresponding restrictions on individuals’ and states’ use and abuse of the good. However, recognizing that obligations follow if global public goods are to be protected is not the same as actually fulfilling—and if necessarily enforcing—such obligations. The harms of failing to protect the environment and antibiotic efficacy are known and therefore, effectively, such goods are recognized, at least in discourse, as primary goods. This is evidenced by a call for global action to protect these goods; for instance, in the Kyoto protocol and the Copenhagen accord as well as in subsequent discussions at Cancun, Durban, Doha and Warsaw; in the ongoing work of the Intergovernmental Panel on Climate Change (most recently its 2014 report); and in global initiatives by the WHO and national governments to protect the efficacy of antibiotics.^11^ These endeavours are, of course, inadequate and the needs—or rather preferences—of states and individuals continue to trump these global public goods. But, although too often practice has not changed significantly, the primary nature of these goods is recognized.

## Global Public Goods and the Health of Migrants

Having laid out why a global public good approach might be interesting and useful for considering the health of migrants (first, it moves the debate from competing individual rights, and second, it provides self-interested reasons for those not convinced by migrant rights arguments) and having described the nature of global public goods, we will in this final section consider what a public goods approach might contribute to the health of migrants. The last section outlined the defining criteria of global public goods. At first glance it might seem unlikely that the health of migrants can be convincingly argued to fit these criteria. Surely the health of migrants can be neglected and they can be refused health goods without *all* individuals being exposed to significant harm? However, while some health care (both as prevention and treatment) could be denied to migrants without exposing others to harm, there are at least some areas where we can argue that protecting migrant health does protect all individuals (and that if migrant health is not protected that individuals will actually suffer preventable harm). It is these we will focus on.

We can begin with the global public good we have just considered, that of antibiotic efficacy. As a global public good then antibiotic use by migrants should be managed to protect antibiotic efficacy; as it should for all. Thus, we have the first health good which should be given to migrants, not because it is their right to have correct antibiotic treatment, but to protect the global public good of antibiotic efficacy and thus to protect all. One reply to this suggestion might be that antibiotic efficacy would be better protected simply by refusing antibiotics to migrants; however, experience in contexts where access to antibiotics, especially for serious conditions, is reduced is that misuse is common. For instance, as discussed in the previous section with regard to antibiotic use in the developing world, in contexts where access is scarce and treatment is correspondingly highly valuable, misuse, in the form of stock-piling and failure to finish courses of treatment, is high. Such behaviour contributes significantly to the increase of antibiotic resistance. Of course, if it were possible to absolutely deny all antibiotics to all migrants, then this might contribute to protecting antibiotic efficacy, but this is unlikely in practical terms, and of course would be exceptionally harmful and require additional, probably coercive measures, to enforce. There are a number of other health goods which might well be offered to migrants on these grounds and we will consider just two more of these; first, the management of infectious disease and second, herd immunity.

First then, managing infectious disease. In an age of pandemics, this is a particularly important global health good, as we are, at the time of writing, six months into the worst Ebola epidemic in history. This health good overlaps with antibiotic efficacy. The risks of infectious disease rise as strains of antibiotic diseases rise and diseases become ever harder to treat and increasingly life-threatening, such as multi-resistant TB, already mentioned above. But, there are other global health goods which follow from considering the significant harms of infectious disease.

In terms of the first criterion, if the global public good of being ‘as free as reasonably possible from infectious disease’ (or some similarly conceived good) is not protected, then *all* individuals (current and future) will be exposed to significant harm (and often will actually suffer harm, harms preventable by the protection of the good). The current Ebola pandemic shows the difficulty of containing such threats locally in areas lacking health infrastructure and health professionals—returning us to the debate at the beginning of the paper regarding the global flow of health workers from the developing world. Such issues are exacerbated by fear and misinformation, and as a result, healthcare workers may fail to report for work or be stigmatized if they do. But, and importantly for global public good claims, epidemics threaten not only the local area, but they are global threats (although predictably the burden falls disproportionally on the poor). This said global concern is greatest when the developed world is threatened. This is shown in the response, or lack of it, to Ebola and was true in the SARS outbreak. This epidemic originated in China on 16 November 2002, but the global response began on 12 March, when the WHO issued a global alert. This was after the first reported case in Canada on 5 March; and a few days post the alert, and on the same day that emergency travel advice was issued (15 March), three ill passengers were taken off a plane traveling from New York to Singapore (WHO, 2003a). While the greatest death toll occurred in China (5327) and the Hong Kong Special Administrative Region (1755), and the highest number of fatalities in the West reaching a fraction of that (250, in Canada) (WHO, 2003b), the response was heightened with the spread of the disease to the West.

Given that disease is no respecter of borders and infectious health threats are global, the first criterion is met, the harms are indeed significant. The fact that the developed world has shown willingness to respond when threatened might be used to support a global public good model with focuses on threats to all and collective action. This brings us to the second criterion; the global public good cannot be protected without collective action (nor can the resulting harms be prevented without collective action). Here collective action could be a large number of interventions including the enforcing of quarantine, the use of protective equipment and protocols, measures to prevent movements of people and global access to health care professionals and to treatment. What is required is significant. In a recent comment on the Ebola crisis, Lawrence Gostin offers a list of what is needed to manage outbreaks of infectious disease including, ‘community, laboratory, public health, and clinical personnel; infection-control equipment, supplied, and protocols; health worker training; laboratory facilities with high biosafety capabilities; health facilities, including safe isolation units; and communication systems that can effectively deliver important public health information’ (Gostin, 2014: 1). To provide such very necessary goods for all, a collective model is needed. One possibility, which Gostin discusses, is a Global Health Emergency workforce (which was proposed by the WHO in 2011 but which was never actualized) (Gostin, 2014). However, whatever approach is taken, clearly collective goods are in question and collective models which can prioritize such goods are likely to be more effective than those which focus on individuals.

As the first two criteria are met, so the normative criteria are invoked; that, this is a primary good which *should* be protected if significant harms to all individuals are to be prevented, and accordingly states and/or individuals cannot be allowed to choose to neglect them. The immediate threat of infectious disease means that protecting this good and preventing the attendant harms is often recognized as globally significant, and perhaps, this is an area in which the need to protect migrants, as part of protecting all, is easy to recognize. This then, is another instance in which a public good approach does result in providing some health goods for migrants. Moreover, these goods might be extensive if one considers what requiring goods such as health infrastructure might entail.

Second then, and more briefly, herd immunity.^12^ Herd immunity is the emergent property of vaccination by which all members of a given community are protected from a specific vaccine-preventable disease by majority participation in vaccination programmes for that disease (Anderson and May, 1985). When the number of vaccinated persons falls below the herd immunity threshold, resurgence of the disease becomes likely. Herd immunity is interesting as a global public good because it relies on collective action of most (not quite all), recognizing that some people—the immunocompromised, the very young and very old—are unable to participate in vaccination programmes for safety reasons. Like the environment and antibiotic efficacy, herd immunity (broadly) fits the criteria outlined above: first, if the public good is not protected, then all individuals (current and future) will be exposed to significant harm; and second, the global public good cannot be protected without collective action (nor can the resulting harms be prevented without collective action). From these then follows the normative requirement, the third criteria, that this good should be prioritized and protected. Unlike antibiotic efficacy, a small number of non-compliant individuals will not destroy the global public good, but even with this caveat, the general case is the same, states must act to protect herd immunity and nearly all individuals must to do the same.

This discussion—of antibiotic efficacy, of managing infectious disease and of herd immunity—provides just a small number of examples of the types of health goods which migrants might be entitled to from a global public good perspective. There are many more goods which could fall into the global public good category, or which could be argued to contribute to the goods we have outlined. For example, it might be possible to make an argument that access to clean water, food and adequate shelter as well as to basic health goods is a global public good, as they vastly reduce the likelihood of the spread of infectious disease and so contribute to protecting a global public good. Alternatively, perhaps, arguments about access to contraception and abortion might be made on global public goods grounds regarding population control. Cashing out the details of exactly what a global public goods account could contribute to the health of migrants would take further discussion. But, while it may not grant the full basket of health goods that a rights-based approach can, it will at least give additional reasons for some health measures to be granted to all migrants and automatically.

## Conclusion

This brief discussion has shown that there are at least some health goods which should be provided to migrants. Providing such goods is justified not on individual rights grounds but on the grounds of the protection of all. This provides a means for all to endorse these goods. At least some basic health goods (in the forms of both treatment and prevention) should be accorded to migrants on global public good grounds. Using this approach denies there is conflict between the rights of one group of individuals and another group of individuals or between the global public good and individuals’ rights. Only together can these goods be protected and the harms to individuals prevented. Conceptualizing it in this way simply removes the claim that the rights (or resources) are in competition on these issues. For any to be protected, all must be protected. Further, it provides a reason for those who do not support the rights of migrants to grant such health goods to them.

As a final comment it is of course the case that these health goods are provided to migrants not because they are migrants but because they need to be provided to all to protect public goods. This of course is the case, but this parallels many migrant rights arguments which use human rights to claim migrants’ rights; again, arguments based on what should be provided to all. This alternative approach should be used separately and together with individual approaches to improve the provision of health goods to migrants.

## Notes

1. Our recent work on non-individual ethical models is in genetics (Widdows, 2013) and in debates about global ethics and health ethics more generally (Widdows, 2007; Widdows and West-Oram, 2013).

2. It is important to note that there is no human right to free movement as such because, while all persons are permitted to leave a territory, sovereign states continue to reserve the power to determine who can enter or stay in their territory. However, the 2003 International Convention on the Protection of the Rights of All Migrant Workers and Members of Their Families seeks to ensure that both illegal and legal migrants and their families are protected in basic and important ways in the host government’s policies (OHCHR, 2014).

3. This language occurs in various media, public and political discourse about immigrants. See, for example, Ana (1999) and Travis and Malik (2013).

4. In 2010, *The Economist* reported that ‘Remittances [in the Philippines] are now equivalent to 11% of the economy’ (Banyan, 2010). In 2014, The World Bank reported that remittances to developing countries are set to increase ‘7.8 per cent over the 2013 volume of $404 billion, rising to $516 billion in 2016’ (The World Bank, 2014). Moreover, they argue that remittances ‘remain a key source of external resource flows for developing countries, far exceeding official development assistance and more stable than private debt and portfolio equity flows. For many developing countries, remittances are an important source of foreign exchange, surpassing earnings from major exports, and covering a substantial portion of imports.’ (The World Bank, 2014).

5. We are not alone in introducing collective approaches into this debate; however, such interventions are relatively few. Significant contributions include those of: Mathias Risse’s paper, which introduces public goods arguments to the immigration debate (although the focus is on population density, natural resources and the United States; Risse, 2008); and Alexander Betts’ paper, which focuses explicitly on public goods and refugee protection, although not on health (Betts, 2003). Cars *et al.* (2008), as discussed in footnote 8 below, also argue for the need for collective action on combating the issue of antibiotic resistance, which should be regarded as a common good.

6. The language of ‘choice’ is used here to emphasize that the goods which are protected legally and in terms of policy norms are open to change and reflect individual and society preferences. We envisage that the protection of such goods could be enforced through a mixture of soft and hard law and policy.

7. Though, of course, there are sceptics about the links between climate change and such adverse effects (for instance, Lomborg, 2001). However, such views are increasingly outside mainstream scientific and political opinion.

8. This paper builds on previous work done by Widdows and Peter West-Oram (Widdows and West-Oram, 2013).

9. Since the introduction of antimicrobial medicines in the 30s (Cohen, 1992; Van Epps, 2006), the threat posed by common, often lethal, and formerly untreatable, diseases has been dramatically reduced (Iseman, 1993; Reichman, 1997).

10. Cars *et al.* (2008), for instance, argue that there is a trend of antibiotic resistance and that while individual stakeholders might be aware of the problem, collective action—from politicians, public health workers and consumers—has been slow partly because responsibility for taking relevant measures does not lie with one body in particular. They press for collective action from national and international leaders, behavioural change from consumers and providers and bodies that are willing to develop antibacterial agents to respond to current public health needs. In this regard, they contend that antibiotics must be regarded as a common good.

11. WHO state that they will continue their support of bodies linking the global environmental and health agendas by providing expertise and advice (WHO, 2014). National government responses vary but a good range of measures is listed in environmental assessments of European countries (European Environmental Agency, 2010).

12. Elsewhere we explore in more details whether herd immunity is a global public good (Widdows and West-Oram, 2013).
